# Supplementary material for: Effectively modulating thermal activated charge transport in organic semiconductors by precise potential barrier engineering
Source: Nat Commun. 2021 Jan 4;12:21. doi: 10.1038/s41467-020-20209-w (PMC7782849; doi:10.1038/s41467-020-20209-w)
Supplement: Supplementary file 1 — Supplementary information [file 41467_2020_20209_MOESM1_ESM.pdf]

Supplementary Information

Effectively Modulating Thermal Activated Charge Transport in Organic Semiconductors by Precise Potential Barrier Engineering

Yinan Huang[1,#], Xue Gong[2,3,#], Yancheng Meng[2,3], Zhongwu Wang[1], Xiaosong Chen[1], Jie Li[1], Deyang Ji[1], Zhongming Wei[5], Liqiang Li[1,4,*] & Wenping Hu[1,4]

Correspondence and requests for materials should be addressed to L.Q.L. (email: lilq@tju.edu.cn)

Table of contents:

1. **The topography of DNTT films with different grain sizes and the corresponding transfer curves**
2. **The output curves of the DNTT OFETs with different grain sizes**
3. **The cycle-scan transfer curves at variable temperature of the OFETs with grain size of 350 nm**
4. **The discussion of temperature coefficient of resister (TCR)**
5. **The fitting plot of ln(J/T^2) as a function of 1000/T of the OFETs with grain size of 200 and 520 nm**
6. **I-V characteristic of the device by two-terminal measurement**
7. **The dependence of mobility on gate voltage**
8. **The estimation of carrier density in the off-state**
9. **The discussion of theorical and experimental values of E_B**
10. **Response of temperature sensor to finger touch**
11. **Mathematical analysis of the change trend of E_{Be} versus l**
12. **Fabrication of pentacene polycrystalline OFETs.**
13. **Universality test of potential barrier engineering**
14. **List of growth and electrical data of DNTT films with different grain sizes**
15. **List of parameters of the devices in different batches.**

1. The topography of DNTT films with different grain sizes and the corresponding transfer curves

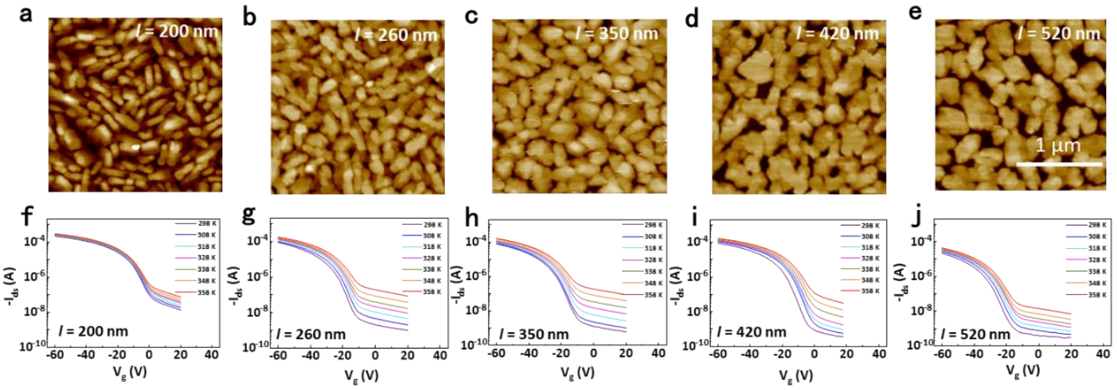

Supplementary Fig. 1 | The topography of DNTT films with different grain sizes and the corresponding transfer curves. a-e, Topography images of DNTT polycrystalline films with grain size of 200 nm, 260 nm, 350 nm, 420nm and 520 nm, respectively. **f-j,** The temperature dependent transfer curves of OFETs with different DNTT grain sizes corresponding **a-e**. The character l in all figures denotes grain size.

By controlling the evaporation conditions, a series of DNTT film with different grain sizes were prepared, and the detailed conditions are displayed in Experimental section and Supplementary Table 1. It was observed by atomic force microscopy that grains were irregularly granulated on the surface of the insulating layer. The grain sizes were measured to be about 200 nm, 260 nm, 350 nm, 420 nm and 520 nm, respectively. The grain size as denoted by averaged length of all grains in 2 μm × 2 μm region. The temperature dependences of transfer curves were measured at drain-source bias of -60 V and gate bias swept from -60 V to 20 V.

In addition, it should be noted that the grain size is measured from AFM morphological image. To ensure the consistence between the grain size on the surface and that in the bottommost layer, thin film (15-20 nm) is used in this

work. Thinner film (about 10 nm) generally leads to discontinuous film, indicating that the bottommost layer does not have bigger grain size to form continuous film. Thicker film (> 25 nm) was also tested, which shows smaller temperature sensitivity. The grain size in thicker film also does not has obvious effect on the temperature dependent charge transport, since the grain size on the surface of thick film may be not the real grain size.

2. The output curves of the DNTT OFETs with different grain sizes

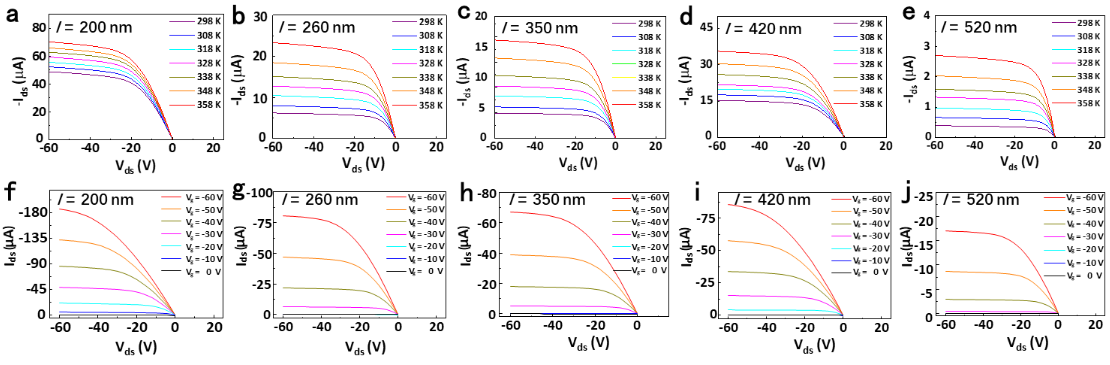

Supplementary Fig. 2 | The output curves of the DNTT OFETs with different grain sizes. a-e, The temperature dependent output curves of OFETs with grain size of 200 nm, 260 nm, 350 nm, 420 nm and 520 nm, respectively. **f-j**, The output curves of OFETs with different DNTT grain sizes at 25 ^{o}C.

3. The cycle-scan transfer curves at variable temperature of the OFETs with grain size of 350 nm

The cycle-scan transfer curves (Supplementary Fig.3) at variable temperature exhibit slight hysteresis, which indicates the good operation stability and slight bias stress and guarantees the credibility of the dependence of carrier transport property on the temperature. Slight bias stress also indicates that there are fewer deep traps on the interface between semiconductor and

insulating layer.

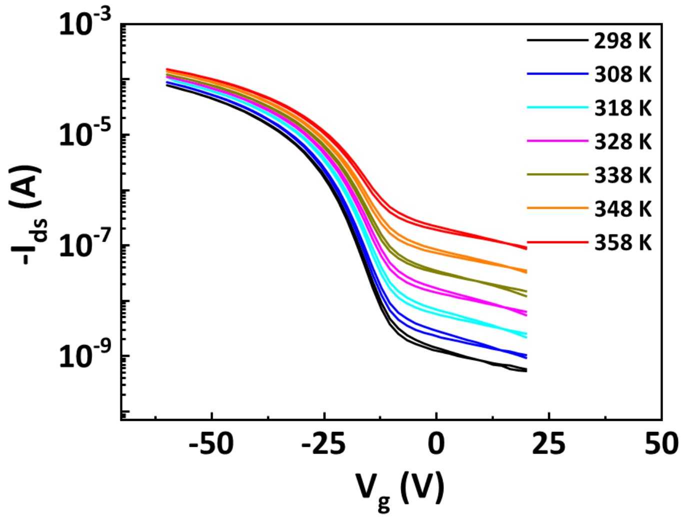

Supplementary Fig. 3 | The cycle-scan transfer curves at variable temperature of the OFETs with grain size of 350 nm.

4. The discussion of temperature coefficient of resister (TCR)

For easy comparison with other kinds of thermosensitive materials or devices, we also calculated the temperature coefficient of resistance (TCR) as sensitivity with Eq. (1).

$$\mathrm{TCR} = \frac{1}{R}\frac{dR}{dT} \times 100\% \tag{1}$$

where *R* is the base resistance at 298K.

5. The fitting plot of ln(J/T²) as a function of 1000/T of the OFETs with grain size of 200 and 520 nm

These results in Supplementary Fig. 4 show a good fit with Eq. (6), indicating that the charge transport in the DNTT films accords with the thermionic emission model. The critical parameter $\frac{E_F - E_C + E_B - qV_B}{k_B}$ can be obtained from the slops of these fitting curves, which determines the degree of temperature

dependence of charge transport. This point is discussed detailly in the Discussion Section of main text.

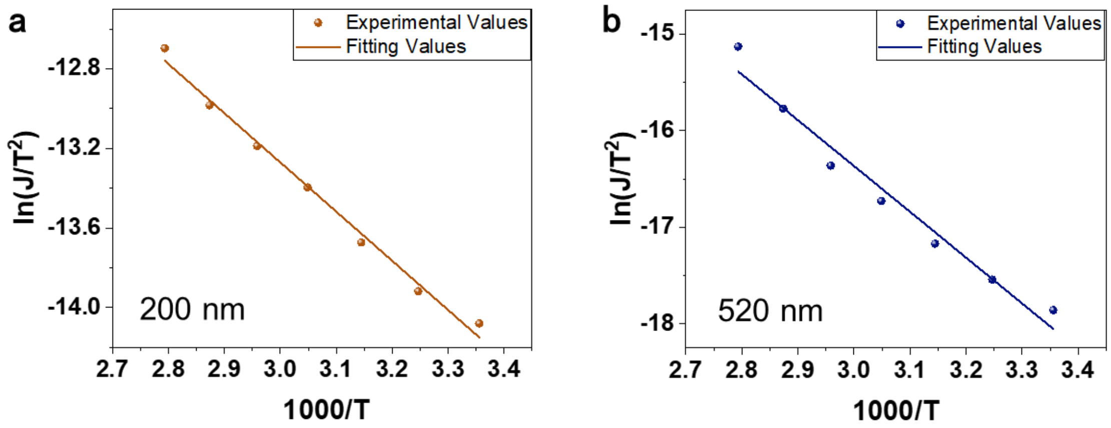

Supplementary Fig. 4 | The fitting plot of ln(J/T^2) as a function of 1000/T of the OFETs with grain size of (a) 200 and (b) 520 nm.

6. I-V characteristic of the device by two-terminal measurement

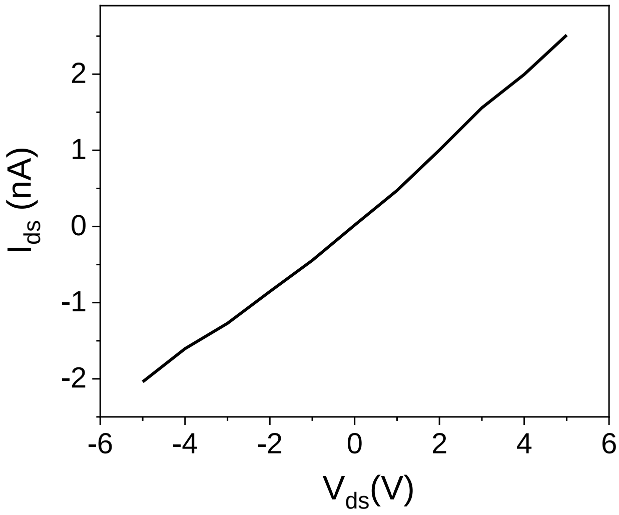

Supplementary Fig. 5 | I-V characteristic of the device by two-terminal measurement.

7. The dependence of mobility on gate voltage

The mobility in saturated region was extracted by field-effect method:

$$I_{ds} = \frac{W}{2L}\mu_{sat}C_i(V_g - V_{th})^2 \quad (2)$$

where W and L are width and length of the conducting channel, and Ci is unit-area capacitance of dielectric layer. DNTT is a thienothiophene small molecule organic semiconductor. These molecules are highly delocalized, closely stacked and a low degree of disorder[1,2]. The researches by Liu suggest that the organic semiconductors with high degree of delocalization and order show week dependence of mobility on gate voltage[3,4]. The dependence of μ and $(V_g - V_{th})$ in this work was examined. As shown in Supplementary Fig. 6, the result indicates the week dependency of μ and V_g, which is consistent with the previous reports. Therefore, μ at V_g-V_{th} = 0 was approximated as μ_{off} for the estimation of carrier density in the off-state.

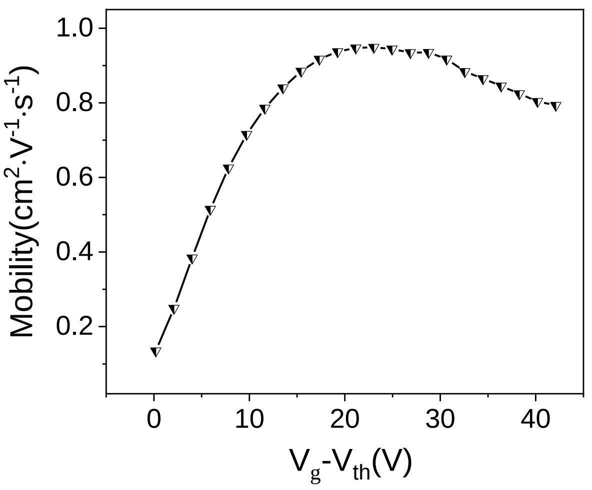

Supplementary Fig. 6 | The dependence of μ on $(V_g - V_{th})$ of the DNTT OFET.

8. The estimation of carrier density in the off-state

The carrier density in the off-state n_{off} was calculated by drift equation $J = qn\mu E$, where q is the electric charge of an electron and E is electric field intensity. According to this equation, the n_{off} of the DNTT devices are estimated to be

10^{13}~10^{14} cm^{-3}, which is reasonable and also accords with the previous reports[5,6]. Although thin film system generally has higher density of impurities than organic single crystal, it shows lower carrier concentration[5-7]. Because only specific impurities may serve as dopant and contribute to formation of free carriers, and most impurities serve as traps or scattering centers in organic semiconductors[8-12]. It should be pointed that the n_{off} here is density of free carriers, and the n for calculating Debye length should be density of total carriers[13-15]. The carriers in off-state is mainly derived from impurities that act as dopants. In our experiment, the DNTT OFETs were fabricated in the similar condition (only evaporation rate and substrate temperature were tuned), so the doping density should be consistent. Nevertheless, the estimation of n_{off} shows decreasing trend with the increase of grain size, which may be explained by the fact that larger barrier at GBs results in more restriction of carriers. In consequence, n_{off} of 200 nm DNTT film with minimal barrier may be closer to total carrier density (n_{tot}). Therefore, it is more reasonable to use this value as an approximation of n_{tot} to calculate Debye length. From the estimation with n_{off} of 1×10^{14} cm^{-3}, the Debye length can be determined to be about 200 nm, which is similar to some reports in semiconductors with low doping level[6,15].

9. The discussion of theorical and experimental values of E_B

In theory, E_B of 350nm DNTT film is estimated to be 9.3 meV by Eq. (2), which is less than the experimental value of 27.7meV by KPFM. It may be due to the following aspects:

(1) The assumption of negligible physical size of grain boundary for theoretical analysis. In fact, the physical size of GBs has complicated effect on potential barrier, and Chwang reported that the larger physical size may result in larger potential barrier[16].

(2) The assumption of identical crystallite neglects the orientation of grains. It is reported that the angle of grains also influences potential barrier[16-18]

(3) The statistics of the grain sizes. In our work, the sizes are artificial statistics from AFM measurement. Although we have tried our best to be as accurate as possible, some errors are inevitable, especially for some irregular grains and indiscernible boundaries.

(4) We used the maximum value of the height of the potential measured by KPFM in this manuscript, which may overestimate the value. If the average value is adopted, the experimental potential by KPFM would be near to the theoretical one.

Although the above factors may cause that the measured barrier values are larger than the theoretical ones, they are in the similar and reasonable range, which can prove the credibility of the theoretical deduction.

10. Response of temperature sensor to finger touch

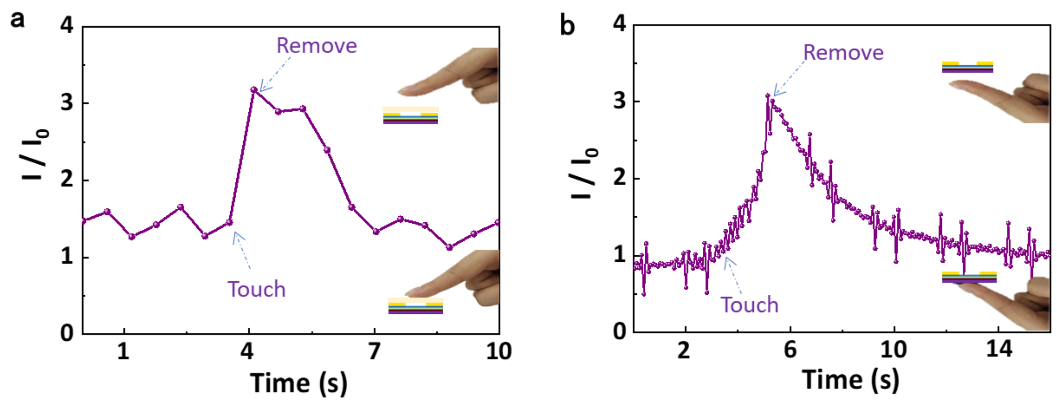

Supplementary Fig. 7 | Response of temperature sensor to finger touch. a, The finger touch on/off the front side of sensor covered with polyimide film (about 0.06 mm). **b**, The finger touch on/off the back side (about 450 μm silicon).

When finger touch on/off the front side of sensor covered with polyimide film (about 0.06 mm), the response and recover time are 0.58 s and 1.76 s, respectively. When finger touch on/off the back side (about 450 μm silicon), the response and recovery are 2.6 s and 8.2 s, respectively. If without the encapsulation or substrate layer, the sensor may show quicker response and recovery speed.

11. Mathematical analysis of the change trend of E_{Be} versus l.

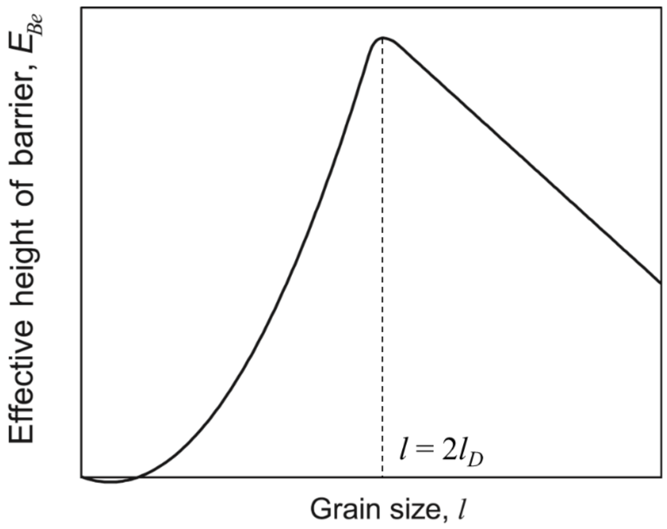

Supplementary Fig. 8 | The change trend of E_{Be} versus l.

The differential sensitivity can be defined as

$$S_D = \frac{1}{J}\frac{dJ}{dT} \tag{3}$$

Inserting Eq (1) $J = A^{*}T^{2}exp\left(-\frac{E_F - E_V + E_B - qV_B}{k_B T}\right)$, we can find that

$$S_D = \frac{2T + \frac{E_F - E_V + E_B - qV_B}{k_B}}{T^2} \tag{4}$$

Obviously, the sensitivity at certain temperature shows positive correlation with $(E_F - E_V + E_B - qV_B)$, which is consistent with the fitting results (Fig. 3e). As we discussed in Theoretical analysis Section, E_F-E_V makes no difference for the devices with the same materials and processes, so the key parameter influencing sensitivity is the effective height of barrier, E_{Be} = $E_B - qV_B$.

According to Eqs. (4) and (5) in main text, the potential barrier E_B and the voltage V_B are competitive for E_{Be} and can be tuned by grain size. If the applied voltage is defined value, V_B always increases lineally with grain size. Supplementary Fig. 7 exhibits two regimes: (1) E_B increases as a quadratic function of l when $l < 2l_D$. In this state, E_{Be} is generally increasing with l. It should be noted that E_{Be} is negative when l is very small, which means the potential barrier is too small to restrain charge carrier. (2) However, when $l > 2l_D$, E_B no longer increases with grain size. While V_B still enlarges, E_{Be} thus begin to decline. Therefore, maximum of E_{Be} (i.e., sensitivity) should be obtained when l is around $2l_D$.

12. Fabrication of pentacene polycrystalline OFETs.

Pentacene and polystyrene (PS) were purchased from Sigma-Aldrich. Polystyrene was dissolved in toluene with a concentration of 10 mg/mL. The PS was spin-coated (3000 rpm, 50 s) onto the surface of SiO_2. Pentacene polycrystalline films were vapor deposited on the insulating layer PS surface below 10^{-4} Pa. The 310 nm and 371 nm grain sizes were deposited at a rate of about 0.005 nm/s, 0.002 nm/s and a substrate temperature of 45 °C, respectively. The 246 nm grain size was deposited at a rate of about 0.015 nm/s, and a substrate temperature of 25 °C. The gold-plated electrodes with a thickness of 20 nm were the source-drain electrodes with a vapor deposition rate of about 0.005 nm/s, and the channel length and width were 50 μm and 1000 μm, respectively.

13. Universality test of potential barrier engineering

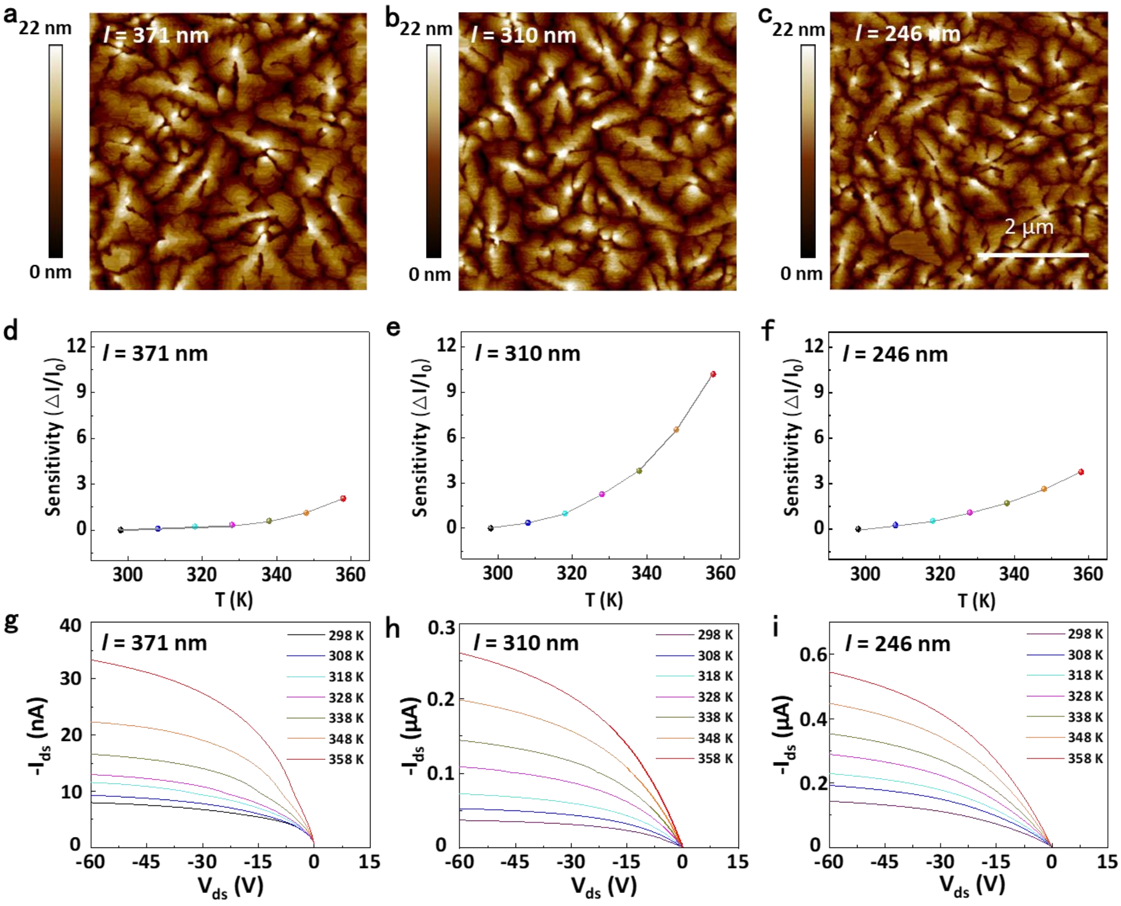

Supplementary Fig. 9 | The topography of pentacene films and characteristic curves of the OFETs with different grain sizes. a-c, Topography images of pentacene polycrystalline films with grain size of 371 nm, 310 nm and 246 nm, respectively. **d-f,** The temperature dependent off-state Green‐Sensitive Phototransistor Based on Solution‐Processed 2D n‐Type Organic Single Crystal currents of OFETs with different grain sizes corresponding **a**-**c**. **g-i.** The output curves of OFETs with different grain sizes corresponding **a**-**c**.

Pentacene films with different grain sizes of about 371 nm, 310 nm and 246 nm were prepared. These devices sustain the steady working status in this temperature range. With increasing grain sizes, the sensitivity shows a peak value at the grain size of about 310 nm.

14. Growth and electrical data of DNTT films with different grain sizes

Supplementary Table 1 | List of growth conditions and electrical data of DNTT films with different grain sizes

Grain Size (nm)	Evaporation Rate (nm/s)	Substrate Temperature (°C)	Sensitivity	Mobility	Δμ/μ*
200	0.01	25	5	1.4	0.22
260	0008	65	90	1.1	0.48
350	0.006	65	155	1.0	0.50
420	0.004	65	80	0.8	0.28
520	0.002	65	21	0.4	0.38

*μ is the saturation region mobility in the on-state

15. List of parameters of the devices in different batches.

Supplementary Table 2 | Summary of the parameters to estimate carrier concentration.

V_{ds} (V)	I_{ds} (A)	L (um)	W (um)	d (nm)	μ_{off} ($cm^2 \cdot V^{-1} \cdot s^{-1}$)
-60	$1.36\times10^{-8} – 4.98\times10^{-9}$	50	1000	20	0.35 – 0.13

I_{ds} and mobility in the off-state of the devices in different batches are extracted, and their values are in the range of 1.36×10^{-8} – 4.98×10^{-9} A and 0.35 – 0.13 $cm^2 \cdot V^{-1} \cdot s^{-1}$, respectively. Therefore, the carrier concentration can be calculated to be 9.8×10^{13} – 1.45×10^{14} cm^{-3} with these parameters.

Supplementary References

1. Illig, S., et al. Reducing dynamic disorder in small-molecule organic semiconductors by suppressing large-amplitude thermal motions. *Nat. Commun.* **7**, 10736 (2016).
2. Peng, B., Huang, S., Zhou, Z., Chan, P. K. L. Solution-Processed Monolayer Organic Crystals for High-Performance Field-Effect Transistors and Ultrasensitive Gas Sensors. *Adv. Funct. Mater.* **27**, 1700999 (2017).
3. Liu, C., et al. A unified understanding of charge transport in organic semiconductors: the importance of attenuated delocalization for the carriers. *Mater. Horiz.* **4**, 608-618 (2017).
4. Yang, T., et al. Understanding, Optimizing, and Utilizing Nonideal Transistors Based on Organic or Organic Hybrid Semiconductors. *Adv. Funct. Mater.* **30**, 1903889 (2019).
5. Horowitz, G. Tunneling current in polycrystalline organic thin-film transistors. *Adv. Funct. Mater.* **13**, 53-60 (2003).
6. Huang, H., Wang, H., Zhang, J., Yan, D. Surface potential images of polycrystalline organic semiconductors obtained by Kelvin probe force microscopy. *Appl. Phys. A* **95**, 125-130 (2008).
7. Kaji, T., Takenobu, T., Morpurgo, A. F., Iwasa, Y. Organic Single-Crystal Schottky Gate Transistors. *Adv. Mater*. **21**, 3689-3693 (2009).
8. Curtin, I. J., Blaylock, D. W., Holmes, R. J. Role of impurities in determining the exciton diffusion length in organic semiconductors. *Appl. Phys. Lett.* **108**, 163301 (2016).
9. Jurchescu, O. D., Baas, J., Palstra, T. T. M. Effect of impurities on the mobility of single crystal pentacene. *Appl. Phys. Lett.* **84**, 3061-3063 (2004).
10. Li, Y., et al. In situ purification to eliminate the influence of impurities in solution-processed organic crystals for transistor arrays. *J. Mater. Chem. C* **1**, 1352-1358 (2013).
11. Street, R. A., Chabinyc, M. L., Endicott, F. Chemical impurity effects on transport in polymer transistors. *Phys. Rev. B* **76**, 045208 (2007).
12. Urien, M., et al. Field-effect transistors based on poly(3-hexylthiophene): Effect of impurities. *Org. Electron.* **8**, 727-734 (2007).
13. Horowitz, G., Hajlaoui, M. E., Hajlaoui, R. Temperature and gate voltage dependence of hole mobility in polycrystalline oligothiophene thin film transistors. *J. Appl. Phys.* **87**, 4456-4463 (2000).
14. Schön, J. H., Kloc, C. Charge transport through a single tetracene grain boundary. *Appl. Phys. Lett.* **78**, 3821-3823 (2001).
15. Singh, B., Singh, J., Kaur, J., Moudgil, R. K., Tripathi, S. K. Thermally and optical induced effects on the structural and electrical parameters of nc-CdS thin films. *J. Mater. Sci-Mater. El.* **27**, 8701-8709 (2016).
16. Chwang, A. B., Frisbie, C. D. Temperature and gate voltage dependent transport across a single organic semiconductor grain boundary. *J. Appl. Phys.* **90**, 1342-1349 (2001).
17. Chwang, A. B., Frisbie, C. D. Field effect transport measurements on single grains of sexithiophene: Role of the contacts. *J. Phys. Chem. B* **104**, 12202-12209 (2000).
18. Weins, M., Gleiter, H., Chalmers, B. Computer Calculations of the Structure and Energy of High-Angle Grain Boundaries. *J. Appl. Phys.* **42**, 2639-2645 (1971).
